# Montreal Cognitive Assessment and Modified Mini Mental State Examination in African Americans

**DOI:** 10.1155/2015/872018

**Published:** 2015-11-04

**Authors:** Kaycee M. Sink, Suzanne Craft, S. Carrie Smith, Joseph A. Maldjian, Donald W. Bowden, Jianzhao Xu, Barry I. Freedman, Jasmin Divers

**Affiliations:** ^1^Department of Internal Medicine (Geriatrics), Wake Forest School of Medicine, Winston-Salem, NC 27157, USA; ^2^Department of Biochemistry, Wake Forest School of Medicine, Winston-Salem, NC 27157, USA; ^3^Department of Radiology, University of Texas Southwestern Medical Center, Dallas, TX 75390, USA; ^4^Department of Internal Medicine (Nephrology), Wake Forest School of Medicine, Winston-Salem, NC 27157, USA; ^5^Department of Biostatistics, Wake Forest School of Medicine, Winston-Salem, NC 27157, USA

## Abstract

*Background*. Sparse data limit the interpretation of Montreal Cognitive Assessment (MoCA) scores, particularly in minority populations. Additionally, there are no published data on how MoCA scores compare to the widely used Modified Mini Mental State Examination (3MSE). We provide performance data on the MoCA in a large cohort of African Americans and compare 3MSE and MoCA scores, providing a “crosswalk” for interpreting scores. *Methods*. Five hundred and thirty African Americans with type 2 diabetes were enrolled in African American-Diabetes Heart Study-MIND, a cross-sectional study of cognition and structural and functional brain imaging. After excluding participants with possible cognitive impairment (*n* = 115), mean (SD) MoCA and 3MSE scores are presented stratified by age and education. *Results*. Participant mean age was 58.2 years (range: 35-83); 61% were female; and 64.9% had >12 years of education. Mean (SD) 3MSE and MoCA scores were 86.9 (8.2) and 19.8 (3.8), respectively. 93.5% of the cohort had a “positive” screen on the MoCA, scoring <26 (education-adjusted), compared with 47.5% on the 3MSE (cut-point < 88). A 3MSE score of 88 corresponded to a MoCA score of 20 in this population. *Conclusion*. The present data suggest the need for caution when applying proposed MoCA cutoffs to African Americans.

## 1. Introduction

With the aging population there is a concomitant diversification of the United States such that, by 2050, it is predicted that the proportion of ethnic minority older adults (≥65) will nearly double (from 19.7% in 2012 to 39.1% in 2050) [1]. Thus, it is critical that cognitive screening tools be useful, applicable, and interpretable in a wide variety of settings and populations.

The Montreal Cognitive Assessment (MoCA) is a freely available screening test of global cognitive function that has been translated into 53 languages (http://www.mocatest.org/) [2]. Though increasingly used, interpretation of MoCA scores in research settings and in diverse populations remains limited by sparse data and lack of comparative data with other tests. The largest source of normative data on the MoCA for US populations comes from the Dallas Heart Study, where 2653 participants were tested [3]. The Dallas Heart Study cohort is ethnically diverse; however, the published data does not provide race-/ethnicity-specific norms based on age or education. This limits the usefulness of the MoCA in minority patients and research participants.

There is also little information regarding how MoCA scores relate to other commonly used cognitive screening tests. Only a few studies have compared the MoCA to the Mini Mental State Examination (MMSE) [4–6], and none, that we are aware of, compared it to the widely used Modified Mini Mental State Examination (3MSE) [7].

The objective of this report is to provide performance data on the MoCA in a large cohort of African Americans and to compare 3MSE and MoCA scores to provide a “crosswalk” for interpreting scores in middle-aged and older African American-Diabetes Heart Study MIND (AA-DHS MIND) study enrollees. These data should help clinicians and researchers interpret MoCA scores in African Americans. They will also be useful for comparing MoCA scores to expected 3MSE scores for clinical assessment and/or inclusion/exclusion criteria in research studies.

## 2. Methods

### 2.1. Participants

The AA-DHS MIND enrolled 530 African Americans with type 2 diabetes (T2D) at the Wake Forest School of Medicine (WFSM). T2D was diagnosed in those with clinical disease onset after 30 years of age in the absence of diabetic ketoacidosis and in the setting of (a) active medical treatment (insulin and/or oral hypoglycemic agents), (b) fasting blood sugar > 126 mg/dL (7 mmol/L) or nonfasting blood sugar > 200 mg/dL (11.1 mmol/L), or (c) hemoglobin A1c (HbA1c) > 6.5%.

### 2.2. Measures/Assessments

Demographic data, medical history, current medications, and vital signs were recorded. Participants self-identified as African American. Participants had a fasting blood draw on which a variety of assays were performed including measures of serum creatinine, blood urea nitrogen, lipids, thyroid stimulating hormone, and vitamin B12 (LabCorp, Burlington, NC). After a morning snack, cognitive testing was performed. This study was approved by the WFSM Institutional Review Board and all participants provided written informed consent.

Cognition was assessed with a 45-minute battery of standard neuropsychological tests chosen to represent a broad variety of cognitive domains, with emphasis on executive function due to the known association between vascular cognitive impairment and executive dysfunction [8]. Global cognition was assessed with the 3MSE [9] and the MoCA [2]. The Rey Auditory Verbal Learning Test (RAVLT) [10] was used to assess learning and memory. Executive function was assessed with the WAIS-III Digit Symbol Coding (DSC) [11] (measuring speed of processing and working memory), the Stroop test [12] (measuring response inhibition), and verbal fluency for animals. Depression was assessed with the Center for Epidemiologic Studies Short Depression scale (CES-D 10) [13, 14]. Interviewers were trained, certified, and subsequently assessed for quality control in all cognitive tests by a single investigator (KMS).

### 2.3. Exclusions for Possible Cognitive Impairment

In an attempt to provide mean (SD) performance data as close to normative data as possible, we excluded participants from the analyses if they had conditions that could cause cognitive impairment or evidence of cognitive impairment on other tests in the neuropsychiatric battery. Participants were excluded from these analyses if they had evidence of hypothyroidism (TSH > 5.0 *μ*IU/L; *n* = 24 excluded), vitamin B12 deficiency (B12 < 147.6 pmol/L; < 200 pg/mL; *n* = 12 excluded), or significant depression (defined as answering with “yes” to “I felt depressed” on 5–7 days of the last week; *n* = 32 excluded). Total CES-D 10 score was not used as a cutoff due to concerns that some of the items have been shown to be invalid in AA [15, 16]. Using published normative data, we also excluded participants who performed worse than 1.5 SD below the mean for their age/education on verbal fluency for animals (*n* = 14 excluded) or the RAVLT long delay (*n* = 45 people). Other tests in our cognitive battery were not able to be used for exclusion purposes because reliable normative data in AA could not be found. In total, 115 participants (of 530) were excluded, leaving a sample size of 415 for these analyses.

### 2.4. Analyses

To determine whether the ordering of tests impacted scores, the first 200 study participants were randomized to receive the 3MSE or the MoCA as either the first or last test in the cognitive battery. Wilcoxon two-sample tests were used to compare the distribution of the observed 3MSE and MoCA scores by randomization order. Poisson-gamma models were also fitted to test for association between these scores and randomization order after adjusting for age, gender, and education level. There was no impact of test order on performance for either the 3MSE or MoCA (*p* value of 0.58 for 3MSE and 0.82 for MoCA; data not shown); therefore, 3MSE was administered first after the 200th participant and all subsequent analyses and presentation of results were done without regard to test order.

Demographic characteristics are presented using descriptive statistics, mean (SD) and %, for continuous and categorical variables, respectively. Means, medians, and SDs are presented for the MoCA and 3MSE by age and education. MoCA and 3MSE scores were compared using the Wilcoxon rank order test. Equipercentile matching was used to create a functional relationship between MoCA and 3MSE. Briefly, a 4th-degree polynomial log-linear equation was used to smooth the observed MoCA and 3MSE scores to reduce irregularities in the score distributions [17]. Equipercentile equating was then performed by determining the percentile rank of a given MoCA score and identifying the corresponding 3MS score that leads to the same ranking. Equipercentile analyses were performed with R-package Equate [18].

## 3. Results

Demographic and health characteristics of the participants are presented in Table 1. Participants had a mean age of 58.2 years (range: 35–83) and 61% were women. Nearly 65% had some college education; 19.8% had at least a four-year degree. The mean (SD) HbA1c was 8.0% (2.0) and the average duration of T2D was 13.0 (7.7) years. Although 83% of the sample had a diagnosis of hypertension, they were under relatively good control with mean (SD) systolic blood pressure of 131.4 (17.7) mm Hg.

*MoCA*. The mean (SD) MoCA score was 19.8 (3.8). Utilizing the <26 cutoff for impairment [2], 93.5% of our participants would be considered impaired after correction for education.  Table 2 provides MoCA score means (SD) and medians by age and education. These scores are not corrected for education, given the fact that we stratified by education. Mean scores decline with age (Spearman's rank correlation = −0.19, *p* < 0.001). For example, amongst those with a high school education (12 years), participants who are less than 55 years old had a mean MoCA score of 20.5 compared to 19.8 for those who are 55 to 65 years old and 18.7 for those who are 65 years old and older. Similarly, scores were higher for those with greater education (Spearman's rank correlation = 0.39, *p* < 0.001), with a 4–7-point spread between those with less than 12 years of education and those with a college degree or more (16+ years), depending on age (Table 2).

*3MSE*. The mean (SD) 3MSE score for the cohort was 86.9 (8.2). Using a cutoff of <88 to define suspected impairment [19, 20], 47.5% of participants would be considered impaired.  Table 3 provides 3MSE means (SD) and medians by age and education. Similar to the MoCA, 3MSE scores were affected by age (Spearman's rank correlation = −0.20, *p* < 0.001) and education (Spearman's rank correlation = 0.41, *p* < 0.001).

**Figure 1:**
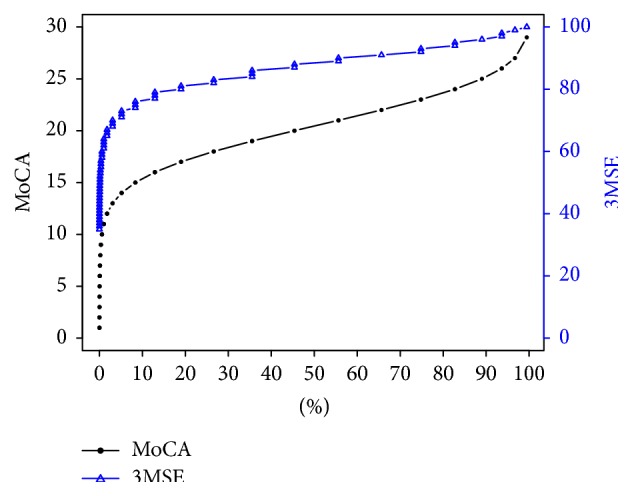
Equivalent MoCA score for a given 3MSE Score. To obtain a MoCA score for a given 3MSE score, find the relevant score on *y*-axis labeled 3MSE and draw a line until it intersects the 3MSE curve and then look down *x*-axis and note the percentile that the score corresponds to. Using that same percentile, draw a line upwards until it intersects the MoCA curve and then note the value on *y*-axis labeled MoCA that this point intersects with. That is the MoCA score that is equivalent to the selected 3MSE score. Reverse the process to find a 3MSE score corresponding to a given MoCA score. For example, 3MSE 80 is approximately the 18th percentile. The 18th percentile MoCA score is approximately 17.

Figure 1 and Table 4 display how the MoCA and 3MSE scores relate using models based on equipercentile performance. For example, a score of 88 on the 3MSE is equivalent to a score of 20 on the MoCA.

## 4. Discussion

This report provides age- and education-stratified data for the MoCA and the 3MSE in a large sample of middle-aged and older-aged African Americans with T2D. As expected, age and education were significantly associated with performance on the MoCA, emphasizing the importance of basing conclusions on stratified data. With these data, clinicians can now compare the MoCA performance of African Americans in their office to mean scores of African Americans of a similar age and education level. This is important, because African Americans are known to perform worse on a variety of cognitive tests than European Americans, potentially due to educational and socioeconomic factors, and thus race-specific normative data are critical for making accurate clinical assessments [21].

With that said, these results should not be considered strictly “normative” data as we did not clinically assess participants for cognitive impairment. When comparing the performance of AA-DHS MIND participants on the MoCA to African Americans in the Dallas Heart Study [3], AA-DHS MIND participants performed several points worse. The mean age of African Americans in the Dallas Heart Study was younger (49.9 years compared to 58.2 years in AA-DHS MIND), with similar mean levels of education, yet the mean (SD) MoCA score was 22 (4). Data specific to African Americans in the Dallas Heart Study were not presented by age/education. The youngest AA-DHS MIND group (age < 55 years) with 13–15 years of education had mean (SD) MoCA scores of 20.7 (3.4). Sixty-two percent of Dallas Heart Study participants fell below the education-corrected cut-point of 26, compared to 93.5% of AA-DHS MIND participants. Performance on the 3MSE was also lower in the AA-DHS MIND sample compared to 3MSE normative data published for a sample of 238 elderly African Americans residing in Tampa, FL [22]. The age strata presented were not the same, limiting direct comparisons between reports. However, mean 3MSE scores in AA-DHS MIND participants who are 55–65 years old were several points lower than those in the participants who are 60–71 years old in the Tampa-based Brown et al.'s study across all education groups but particularly for participants with high school or less education.

There are several reasons why AA-DHS MIND MoCA and 3MSE scores may have been lower than those in published norms. First, all participants have T2D and diabetes has been shown to adversely impact cognition [23]. Second, there may be regional variation in cognitive performance among African Americans, such that those residing in North Carolina may perform worse than those in other regions of the country due to differences in cognitive risk factors (North Carolina is in the stroke belt) [24] or quality of education, especially in the southern US where older participants may have been educated in segregated, rural schools [21, 25]. There are other methodological considerations to factor in when interpreting the current results, including the fact that this was a single center study and results may not generalize to all African Americans based on presence of T2D. However, African Americans have a lifetime risk for T2D that exceeds 50% [26]. These data should not be interpreted as strictly “normative” since no clinical evaluation to determine cognitive status (normal or impaired) was performed. Therefore, we did not provide ROC curves with suggested “cutoffs” for the MoCA as a screening test in African Americans. However, we did exclude participants with hypothyroidism, vitamin B12 deficiency, and depressed mood as all of these conditions could have impacted test scores negatively. In addition, we excluded participants with evidence of memory impairment and poor category fluency for animals in a further attempt to provide data on participants that could be considered free of significant cognitive impairment. In addition, these participants volunteered to be in a research study and study volunteers are typically healthier than the general population (healthy volunteer effect).

In spite of these methodological limitations, there are several noteworthy strengths. We have presented the first report of MoCA scores stratified by age and education in a large sample of African Americans. In addition, we present the first “crosswalk” that can be used to compare scores between the MoCA and 3MSE. This is important because many large epidemiologic studies (examples include the Cardiovascular Health Study, Health ABC Study, Cache County Study, Women's Health Initiative, and Canadian Study of Health and Aging) and clinical trials (Women's Health Initiative Memory Study and Lifestyle Interventions and Independence for Elders (LIFE) Study) have used the 3MSE. In order to compare results in those studies with the more contemporary MoCA test, development of a crosswalk is necessary. This allows researchers to identify a MoCA score that would be comparable to a given 3MSE score for inclusion into or exclusion from a study based on previous studies and allows clinicians to translate risk associated with a specific 3MSE score to a MoCA score that they obtained in clinic in African Americans.

In conclusion, the present data suggests the need for caution when applying proposed MoCA cutoffs to African Americans. Based on the suggested cutoff of <26, 93.5% of AA-DHS MIND participants would have been classified as impaired, which is clearly unlikely. Additional studies using large, diverse populations, where MoCA scores can be stratified by race, age, and education, are needed. We also recommend that MoCA/3MSE comparisons be replicated in other cohorts to determine if our “crosswalk” of scores is applicable to other race/ethnicity groups.

## Figures and Tables

**Table 1:** Characteristics of 415 African Americans with type 2 diabetes mellitus.

Variable	Mean (SD) or %	Range
*Demographics*		
Age (years)	58.2 (9.3)	35–83
Female	61.3	
Education (years)		
<12	10.8	
12	24.3	
13–15	45.1	
≥16	19.8	
*Health related characteristics*		
Diabetes duration (years)	13.0 (7.7)	1.2–56.6
Body mass index (kg/m^2^)	35.9 (8.8)	17.9–75.7
Systolic blood pressure (mm Hg)	131.4 (17.7)	78.5–194.0
Diastolic blood pressure (mm Hg)	76.9 (11.2)	48.0–110.0
Smoking		
Never	46.7	
Past	31.0	
Current	22.3	
Hypertension (%)	83.3	
Fasting glucose (mmol/L)	8.3 (3.6)	2.8–25.1
Hemoglobin A1c (%)	8.0 (2.0)	4.7–15.9
Serum creatinine (*μ*mol/L)	88.4 (26.5)	40.7–388.1
Thyroid stimulating hormone (*μ*IU/L)	1.7 (1.0)	0.01–4.98
Vitamin B12 (pmol/L)	487.1 (272.9)	152.8–1476
*Cognitive function*		
MoCA (0–30)	19.8 (3.8)	6–29
3MSE (0–100)	86.9 (8.2)	46–100
RAVLT delayed recall (0–15)	6.1 (2.9)	1–15
DSC (0–133)	51.1 (16.2)	2–96
Verbal fluency for animals (0–undefined)	16.1 (4.5)	8–31
Stroop interference (seconds)	37.1 (16.4)	−20–134

MoCA, Montreal Cognitive Assessment; 3MSE, Modified Mini Mental State Examination; RAVLT, Rey Auditory Verbal Learning Test; DSC, Digit Symbol Coding.

**Table 2:** Montreal Cognitive Assessment (MoCA) scores by age and education in 414 African Americans.

Age (years)	Education								All	
	<12 years		12 years		13–15 years		16+ years			
	*N*	Mean (SD) median	*N*	Mean (SD) median	*N*	Mean (SD) median	*N*	Mean (SD) median	*N*	Mean (SD) median
<55	15	18.8 (3.1) 19.0	50	19.3 (3.9) 19.5	66	20.7 (3.4) 20.0	28	23.4 (3.1) 24.0	159	20.5 (3.8) 20.0
55–65	15	18.0 (3.0) 18.0	38	18.4 (3.7) 18.5	74	19.8 (3.3) 20.0	36	22.3 (3.6) 23.0	163	19.8 (3.7) 20.0
>65	15	14.4 (3.3) 15.0	13	17.7 (4.2) 19.0	46	19.2 (2.9) 19.5	18	21.6 (3.1) 21.0	92	18.7 (3.9) 19.0
All	45	17.1 (3.6) 17.0	101	18.7 (3.8) 19.0	186	19.9 (3.3) 20.0	82	22.5 (3.4) 23.0	414	19.8 (3.8) 20.0

Note: MoCA scores are raw scores, with no extra point given for education ≤12 years. *N* = 414, not 415, because age was missing for one person. Range for age <55 is 35–54; range for >65 is 66–83 years. For participants with <12 years of education, 11 had 5–8 years and 34 had 9–11 years.

**Table 3:** Modified Mini Mental State Examination (3MSE) scores by age and education in 414 African Americans.

Age (years)	Education								All	
	<12 years		12 years		13–15 years		16+ years			
	*N*	Mean (SD) median	*N*	Mean (SD) median	*N*	Mean (SD) median	*N*	Mean (SD) median	*N*	Mean (SD) median
<55	15	82.7 (6.7) 85.0	50	86.6 (7.2) 88.0	66	89.0 (6.0) 89.5	28	94.2 (4.6) 95.0	159	88.5 (7.0) 89.0
55–65	15	79.7 (10.8) 84.0	38	84.1 (6.8) 84.0	74	87.9 (7.5) 89.0	36	90.1 (6.9) 92.0	163	86.7 (8.1) 87.0
>65	15	73.9 (11.0) 74.0	13	81.1 (8.2) 82.0	46	86.4 (6.6) 86.0	18	90.2 (8.0) 92.0	92	84.4 (9.4) 88.0
All	45	78.8 (10.2) 79.0	101	84.9 (7.4) 86.0	186	87.9 (6.8) 88.0	82	91.5 (6.7) 94.0	414	86.9 (8.2) 88.0

Note: *N* = 414, not 415, because age was missing for one person. Range for age <55 is 35–54; range for >65 is 66–83 years. For participants with <12 years of education, 11 had 5–8 years and 34 had 9–11 years.

**Table 4:** Conversion table between observed MoCA and 3MSE scores in African Americans.

MOCA	3MSE
1	0–37
2	38–40
3	41-42
4	43-44
5	45-46
6	47–49
7	50–52
8	53-54
9	55–57
10	58–60
11	61–64
12	65–67
13	68–70
14	71–73
15	74–76
16	77–79
17	80-81
18	82-83
19	84–86
20	87-88
21	89-90
22	91
23	92-93
24	94-95
25	96
26	97-98
27	99
29	100
